# Comparison of consecutive and restained sections for image registration in histopathology

**DOI:** 10.1117/1.JMI.10.6.067501

**Published:** 2023-11-30

**Authors:** Johannes Lotz, Nick Weiss, Jeroen van der Laak, Stefan Heldmann

**Affiliations:** a Fraunhofer Institute for Digital Medicine MEVIS, Lübeck, Germany; b Radboud University Medical Center, Department of Pathology, Nijmegen, The Netherlands; c Linköping University, Center for Medical Image Science and Visualization, Linköping, Sweden

**Keywords:** computational pathology, image registration, histopathology, multiplexing, machine learning

## Abstract

**Significance:**

Although the registration of restained sections allows nucleus-level alignment that enables a direct analysis of interacting biomarkers, consecutive sections only allow the transfer of region-level annotations. The latter can be achieved at low computational cost using coarser image resolutions.

**Purpose:**

In digital histopathology, virtual multistaining is important for diagnosis and biomarker research. Additionally, it provides accurate ground truth for various deep-learning tasks. Virtual multistaining can be obtained using different stains for consecutive sections or by restaining the same section. Both approaches require image registration to compensate for tissue deformations, but little attention has been devoted to comparing their accuracy.

**Approach:**

We compared affine and deformable variational image registration of consecutive and restained sections and analyzed the effect of the image resolution that influences accuracy and required computational resources. The registration was applied to the automatic nonrigid histological image registration (ANHIR) challenge data (230 consecutive slide pairs) and the hyperparameters were determined. Then without changing the parameters, the registration was applied to a newly published hybrid dataset of restained and consecutive sections (HyReCo, 86 slide pairs, 5404 landmarks).

**Results:**

We obtain a median landmark error after registration of 6.5  μm (HyReCo) and 24.1  μm (ANHIR) between consecutive sections. Between restained sections, the median registration error is 2.2 and 0.9  μm in the two subsets of the HyReCo dataset. We observe that deformable registration leads to lower landmark errors than affine registration in both cases (p<0.001), though the effect is smaller in restained sections.

**Conclusion:**

Deformable registration of consecutive and restained sections is a valuable tool for the joint analysis of different stains.

## 1 Introduction

In histopathology, much insight into disease subtyping, biomarker discovery, and tissue organization is gained by analyzing differently stained histological sections. For this procedure, a fixed tissue is transferred into a paraffin block and cut into 2 to 5  μm thin slices. These slices are subsequently stained by, e.g., immunohistochemistry, and—in a digital workflow—scanned to obtain a digital whole slide image.^1^ The resulting image can be used for digital analysis in biomarker discovery by combining two or more different stains.^2^ Deep-learning models are increasingly used to analyze histopathology slides and the first methods have been cleared for clinical use.^3^ These methods require a large amount of annotated images to learn specific tissue properties. Image registration is used to automatically create annotations as training data in order to reduce the time spent on manually annotating slide images.^4^^,^^5^

Enabled by digital slide scanners, a restaining approach, which was initially used in fluorescence microscopy and known as tissue-based cyclic immunofluorescence (t-CyCIF),^6^^,^^7^ is gaining popularity in bright-field imaging.^4^^,^^8^^,^^9^ Instead of staining consecutive sections and scanning them later, a section is stained and scanned first. In a second step, the stain is washed or bleached, and another stain is applied. After rescanning, both images contain the same tissue with different staining, so that it is possible to compare the same cell with respect to different antibodies or markers. However, we still observe nonlinear deformations in the tissue, which are most likely due to the chemical reactions during the restaining process.

Independently of the sectioning method, researchers face a number of questions when applying image registration to a new dataset. These include as follows.

• Which image resolution is best suited to obtain the best accuracy while keeping the computational cost as low as possible?
• Is deformable registration required or is an affine registration sufficient, especially for the registration of restained sections where little differences are expected between both images?

As we show in this work, image registration is required in both, consecutive and restained image pairs. We compare the accuracy of the registration for both types of image pairs in one single dataset. To our knowledge, no such direct comparison has been made. Various methods and analyses have been published on the registration of consecutive^10^ sections but only recently have a few publications addressed restained sections.^11^^,^^12^ In the case of registration of restained sections, accuracy is achieved at the nucleus level. In the case of consecutive sections, this level of accuracy cannot usually be reached due to the lack of corresponding objects at the appropriate resolution caused by the slice thickness or distance. Here, a good registration of structures with a size above the nucleus level can be achieved based on images with relatively low resolution and the use of a nonlinear deformation model.

We compare the two types of image pairs using the same optimization-based image registration method that is based on minimizing an energy functional consisting of a distance measure and a regularizer.^13^ This class of optimization-based methods is widely used in medical imaging^14^^,^^15^ and has also been applied to problems in pathology.^10^^,^^16^^–^^19^

These energy-minimizing methods make explicit model assumptions through the choice of distance measure and regularization scheme. When applying a method to a new dataset, model refinements can be made by adjusting the model’s parameters. For example, when a new dataset contains larger deformations, the weight that balances image distance and regularization can be adapted to allow for larger displacements.

Another class of methods that gained popularity in recent years is based on training a deep-learning model to estimate the deformation in problems in medical imaging^20^ and specifically pathology.^21^ Here the model assumptions are made implicitly by the training data. This in turn makes generalization and adaption to unseen datasets more challenging, although recent work^22^^–^^24^ addresses this issue.

Below, we first describe the registration method and its application to restained and consecutive slide images. We then describe an evaluation framework based on landmark accuracies on two datasets, the “Automatic Nonlinear Histological Image Registration (ANHIR)^17^^,^^25^ Challenge” and on a new dataset “Hybrid Restained and Consecutive Data (HyReCo)”^26^ that contains both consecutive and restained slides and that we make publicly available. Finally, we analyze the accuracy of the image registration method with respect to image resolution and sectioning in both datasets.

## 2 Fully Automatic Image Registration

We compared the registration of the two sectioning methods based on a three-step, energy-minimizing registration pipeline. It consists of (1) a robust prealignment, (2) an affine registration computed on coarse resolution images, and (3) a curvature-regularized deformable registration. The method is based on the variational image registration framework first described by Fischer and Modersitzki,^13^^,^^27^ which has been applied to many clinical fields from histology^28^ to radiology.^29^^,^^30^ A more detailed and formal description of the method can be found in Appendix A.

We used the normalized gradient fields (NGF) distance measure^31^ in all three pipeline steps as it has been shown to be robust to different stains and is suitable for multimodal image registration of histological images.^4^

The NGF distance measure is based on structural changes expressed through the image gradient, and therefore, color information is of limited value. To reduce the amount of image data to be handled, all images were converted from color to gray scale and inverted to obtain a black background while loading from disk.

Images were assumed to be available in a multilevel image data format to reduce the time and memory requirements to load the image data at a given resolution.

All three following registration steps rely on the edge parameter ε, the number of levels $N_{\mathrm{level}}$ of the image pyramid, and the image resolution at the finest level (see Appendix A). The parameters were set independently for each step, such that the registration error was minimal, and the deformation grid was regular in the sense that it was not folded in the image domain. These parameters are shown in Table 1.

**Table 1:** Parameters used in the registration pipeline for all datasets.

Registration step	Parameter values
Step 1: Prealignment	
No. of levels $N_{\text{level}}$	4
No. of rotations $N_{\mathrm{rot}}$	32
Image resolution (μm/px)	∼200
Image size (px)	∼100×200
NGF ε	0.1
Step 2: Affine	
Image resolution (μm/px)	∼248 to 1
Image size (px)	∼100×200 to 25k × 55k
No. of levels $N_{\mathrm{level}}$	3 (248 μm/px) − 11 (1 μm/px)
NGF ε	0.1
Step 3: Deformable	
Image resolution (μm/px)	∼248 to 1
Image size (px)	∼100×200 to 25k × 55k
No. of levels $N_{\mathrm{level}}$	3 (248 μm/px) − 11 (1 μm/px)
NGF ε	1.0
Regularizer weight α	0.1
Control point grid m	257 × 257 nodes

### 2.1 Step 1: Automatic Rotation Alignment

Before histological images were scanned, the tissue was manually cut, preprocessed, and stained in a pathology lab.

After this process, some neighboring tissue slices can end up in arbitrary positions on the object slide (such as upside down or turned in various ways). In general, no assumptions can be made on the initial tissue positioning and—in a first step—we aimed to find a rigid alignment, correcting for global translation and global rotation.

To compute the rigid alignment, the center of mass^32^ of both images was determined using the gray values of the pixels as the weights. To find the best rotation, an exhaustive search was performed in the parameter space with equidistant angles. At each angle, a rigid registration was calculated to optimize the NGF-distance. The angle with the smallest image distance was selected as an initial guess for the subsequent affine registration.

### 2.2 Step 2: Affine Registration

In a second step, again an NGF-based image registration was computed. To allow for additional degrees of freedom, the registration was optimized with respect to an affine transformation and based on a finer image resolution (Table 1).

### 2.3 Step 3: Deformable Registration

In the final step, a deformable image registration based on curvature regularization was computed. The displacement was discretized using a nodal grid of control points. The grid’s resolution was chosen to be coarser than the image resolution (Table 1). Linear interpolation was used to evaluate the deformation between its grid nodes.

## 3 Evaluation

We compared both image acquisition methods with respect to the accuracy of the registration in a new, previously unpublished dataset, HyReCo,^26^ that combines restained and consecutive sections. To relate to the previous work in registration of consecutive sections, we additionally calculated the registration accuracy in the training part of the ANHIR challenge data^33^ evaluating the absolute registration error that was not part of the original challenge.

We reported the distribution of the target registration error ${\left\|r_{k}-t_{k}\right\|}_{2}$, $k=1,\ldots ,N_{\text{images}}$ and its median:

$$\mathrm{MTRE}={\text{median}}_{k}({\left\|r_{k}-t_{k}\right\|}_{2}),$$

over all $N_{\text{images}}$ image pairs and over all available landmarks $r_{k},t_{k}\in R^{2}$ in both datasets.

Histological images, due to their large size, are typically stored at different image resolutions in a pyramid-like image format. This format allows easy access to tissue structures at different scales. Image registration was applied at different scales, and linear interpolation was used to apply the deformation to the images and landmarks, regardless of the original image resolution. Assuming that image resolution affects both the speed and accuracy of registration, we evaluated the registration accuracy at different resolutions.

In addition, a comparative analysis was performed between affine and deformable registration applied to consecutive and restained sections.

To avoid overfitting the registration parameters (Table 1) to the HyReCo data, we directly applied the parameters determined in the ANHIR challenge without specific refinement to the HyReCo images. Moderate modifications in NGF ε, regularizer parameter α, size of the deformation grid and number of levels only show a small influence on the accuracy when registering coarse image resolutions (up to ∼4  μm/px). On higher image resolutions, the parameter choice seems to have a larger impact.

For the comparative statements, statistical tests were performed comparing the distributions of the distances of paired annotations. Normality was rejected for all distributions after the Shapiro–Wilk test. As the HyReCo dataset consists of paired data samples, we selected the Wilcoxon signed-rank test. In the specific case of comparisons between independent HyReCo and ANHIR datasets, the Wilcoxon rank-sum test was used. To address the multiple comparisons problem, we applied the Bonferroni correction. In all comparative statements p-values <0.05 were designated as significant and reported as p<0.05, p<0.01, and p<0.001. Whenever the Bonferroni correction was applied, we made sure to report the adjusted significance level, e.g., p-value $<0.05/N_{B}$ with $N_{B}$ indicating the number of tests.

The registration was applied to consecutive and restained sections in the HyReCo dataset.

### 3.1 Hybrid Restained and Consecutive Data

The HyReCo dataset contains tissue of the human tongue with small oral tumors, which were acquired at the Radboud University Medical Center, Nijmegen, The Netherlands. (The requirement for ethical approval was waived by the IRB of Radboudumc, Nijmegen, The Netherlands, under file number 2020-6972.). All sections were cut with a thickness of 4  μm.

#### 3.1.1 HyReCo subset A (restained and consecutive)

**Fig. 1:**
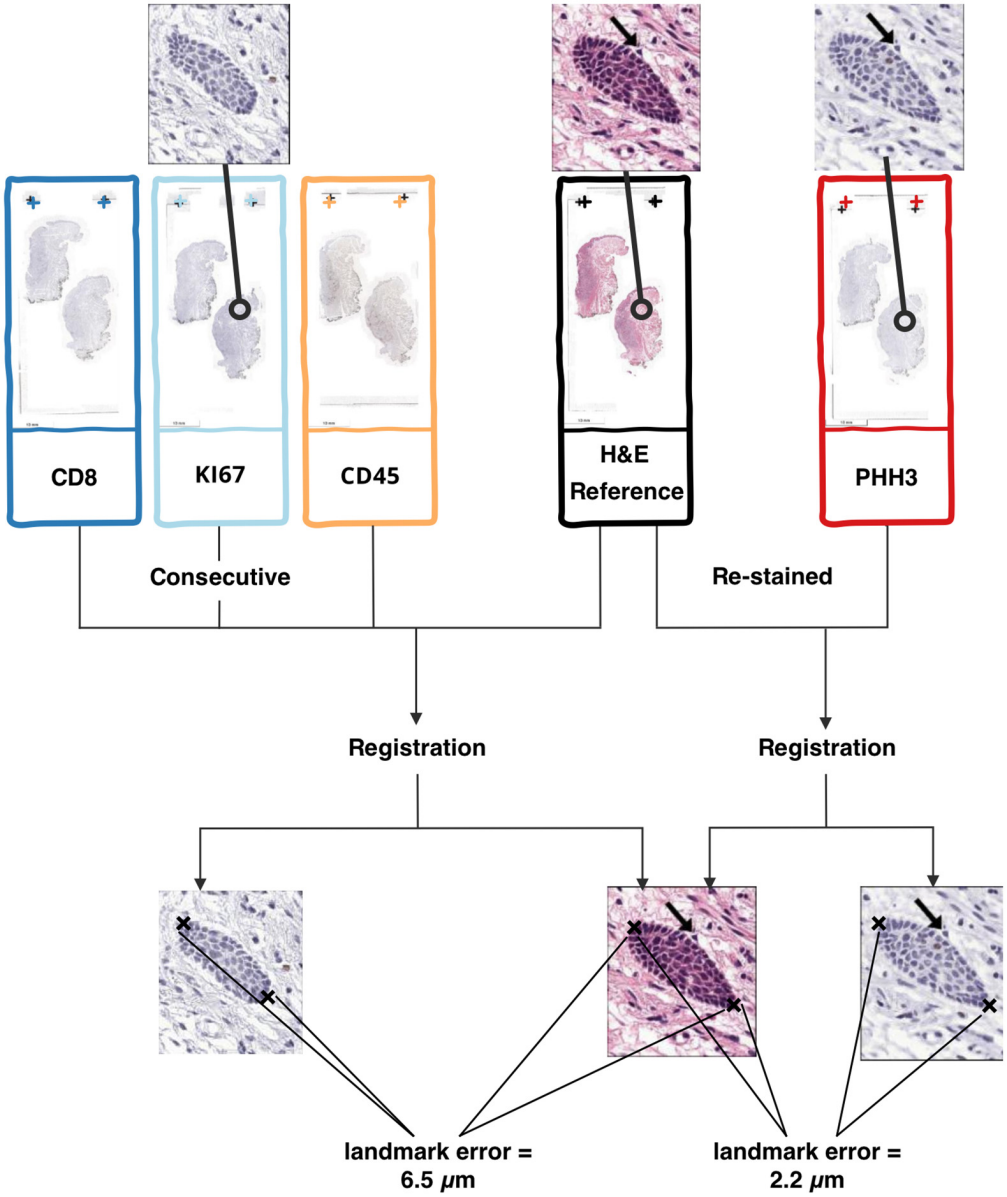
Overview of the comparison between the registration of consecutive and restained sections. Consecutive sections were stained with H&E, CD8, CD45RO, and Ki67. Furthermore, the H&E-stained samples underwent restaining with PHH3 and were subsequently rescanned. Registration between restained sections results in a lower landmark error than between consecutive sections.

**Fig. 2:**
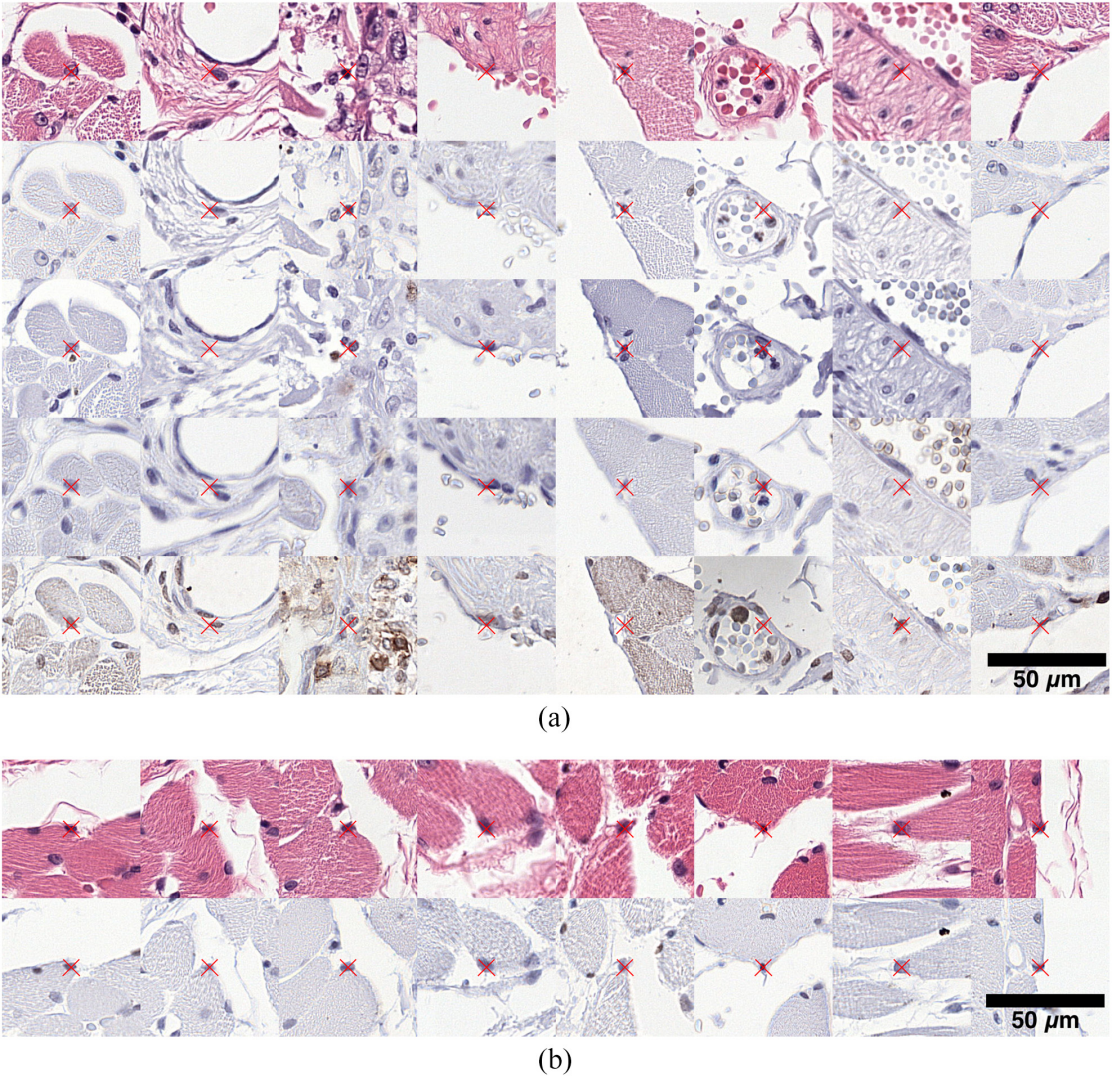
Examples of manually placed landmarks in consecutive and restained sections for registration accuracy evaluation. Each column represents the same position in multiple sections. Stains in rows from top to bottom: (a) HyReCo subset A: H&E, PHH3, Ki67, CD8, and CD45 (b) HyReCo subset B: H&E and PHH3.

It consists of two subsets of slides, first (A) nine sets of consecutive sections, each containing four slides stained with H&E, CD8, CD45RO, Ki67, respectively. In addition, PHH3-stained slides were produced by removing the cover slip from the respective H&E-stained slide, bleaching the H&E stain, restaining the same section with PHH3, and scanning it again, similar to the t-CyCIF technique^6^^,^^7^ that is well established in fluorescence imaging (Fig. 1). For each of these sections, 11 to 19 landmarks (138 per stain, 690 in total) were placed manually at the center or at the boundary of corresponding nuclei or at visually similar structures [see Fig. 2(a) for examples]. Finding the same points across several consecutive slides is challenging, because the same structure is often not present in distant sections. To avoid bias toward a specific stain, all sections of a case were visualized at once on the screen and annotators were asked to identify landmarks where correspondence can be found in all five sections. In some cases, the corresponding position was estimated by comparing the neighboring morphology. All landmarks were reviewed by two experienced researchers.

To estimate a lower bound for landmark accuracy, two researchers annotated the same structures (∼20 landmarks each) in the same and in a consecutive slide. The annotations were made independently of each other and on the same type of structures used in the landmark error calculation.

#### 3.1.2 HyReCo subset B (restained)

To overcome the limitations in annotation accuracy imposed by the simultaneous annotation of consecutive and restained slides, a second subset (B) of restained slides without corresponding consecutive sections were scanned and annotated. An image pair of restained sections contains the same cells and nuclei such that a one-to-one correspondence can be found for most structures. An additional 2357 annotation pairs were produced for 54 additional image pairs of H&E-PHH3 [∼43 annotation pairs per image pair, see Fig. 2(b) for examples]. These have again been verified by two experienced researchers.

All images have been digitized with a resolution of 0.24  μm/px and are ∼95,000×220,000  pixels in size at their highest magnification level.

The dataset including the landmarks has been made available^26^ under the Creative Commons Attribution-ShareAlike 4.0 International license.

### 3.2 ANHIR Dataset

The accuracy of the registration of serial sections depends on the distance between the sections and on the quality of the tissue sectioning. To broaden the scope of the analysis and to make the results comparable to previous work in registration of serial sections, we additionally re-evaluated the accuracy of the registration of the ANHIR challenge data.^33^ Instead of the relative registration error from the challenge, we computed the registration error in absolute values based on the pixel sizes given in the challenge publication.^17^

**Fig. 3:**
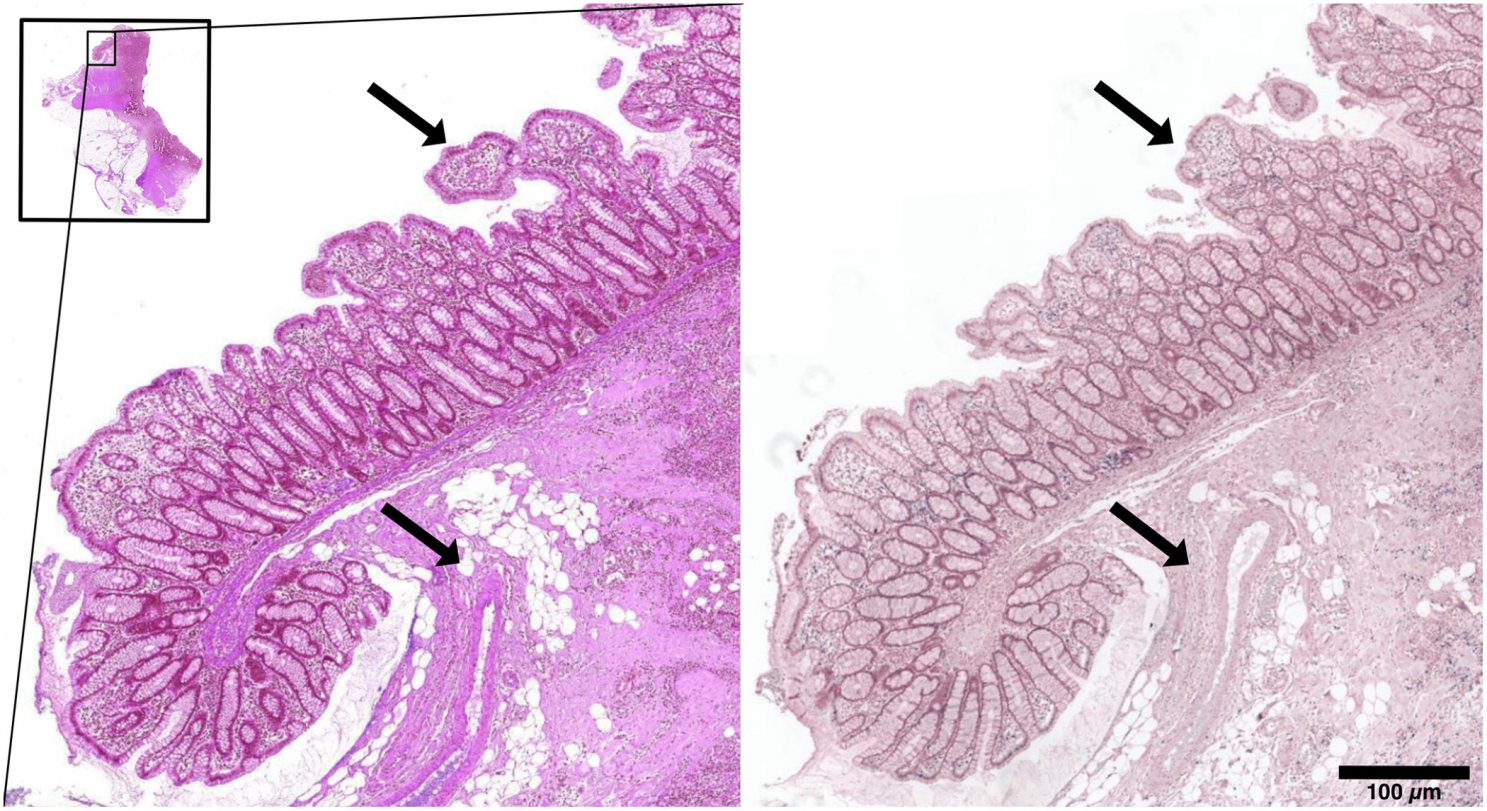
Two consecutive slides from the ANHIR dataset (image set COAD 03). Structures that are only present in one image and that cannot be aligned by image registration are indicated by arrows.

The public part of the ANHIR challenge dataset consists of 230 image pairs from 8 different tissue types (lung lesions, whole mice lung lobes, mammary glands, mice kidney, colon adenocarcinoma, gastric mucosa and adenocarcinoma, human breast, and human kidney) with 18 different stains. For a subset of the ANHIR dataset, the slices are reported to be cut with a distance of 3  μm, for the remaining sections, no thickness is given. An example of the images is shown in Fig. 3. One subset (mammary glands, 38 image pairs) was excluded as the computed pixel sizes were inconsistent with the image sizes.

In the following sections, we measure the accuracy of deformable and affine registration with respect to image resolution on both datasets. We distinguish restained and consecutive sectioning and determine the possible alignment accuracies in the different datasets.

## 4 Results

We applied the three-step registration pipeline to the ANHIR training dataset where the registration hyperparameters were established and—without further parameter tuning—to the HyReCo datasets to evaluate the effects of the image resolution and of the type of registration.

The registration error is computed using manually placed landmarks as described above. The interobserver error on the same section was 0.57  μm±0.36 (mean ± standard deviation), corresponding to 2.3  pixels±1.5 and the intraobserver error was in a similar range (0.53  μm±0.32). In two consecutive sections (H&E and Ki67), the interobserver error was 1.1  μm±0.6 (4.7  pixels±2.6).

### 4.1 Experiment 1: Image Resolution

We measure the registration accuracy with respect to the image resolution used for registration and compare affine and deformable registration on consecutive and restained sections.

#### 4.1.1 Consecutive sections

**Fig. 4:**
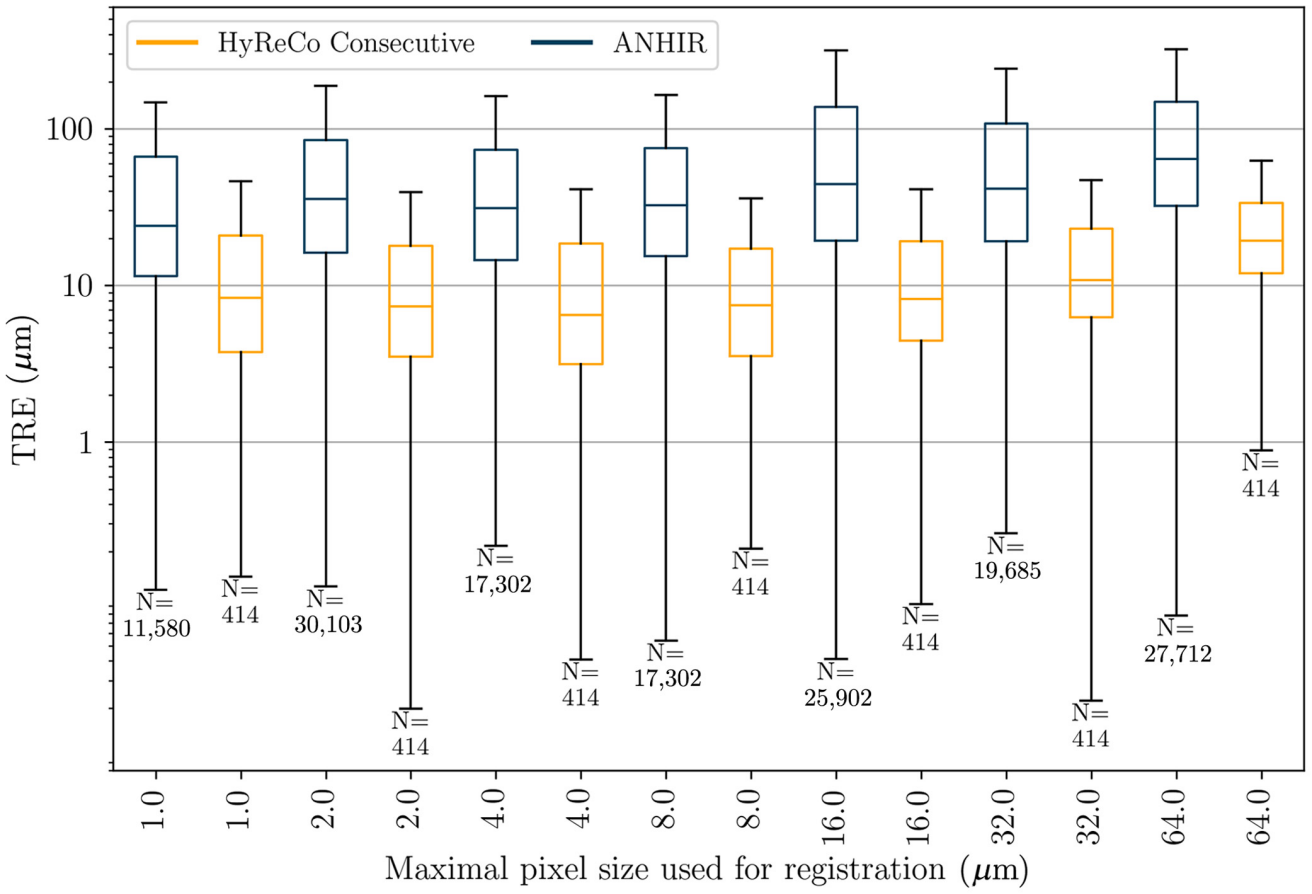
TRE after consecutive deformable registration at different image resolutions for the HyReCo (yellow) and the ANHIR dataset (blue). Compared to the ANHIR dataset the HyReCo dataset shows a smaller registration error for each image resolution ($p<0.001/N_{B}$ after Bonferroni correction by the number of image resolution pairs $N_{B}=6$). The boxes denote the interquartile range and the whiskers extend this range by a factor of 1.5. Variation in N for the ANHIR boxplots is due to limited resolution availability and the limited image size at course resolutions for some slides to which our multilevel image registration could not be applied.

The resulting landmark errors after applying the full three-step registration to the consecutive HyReCo subset A and to the ANHIR dataset are shown in Fig. 4.

Comparing different image resolutions, smaller pixel sizes lead to a smaller registration error up to a level of saturation that differs between datasets. In the HyReCo dataset, we observe improvements for resolutions up to 8  μm/px. No improvements can be shown for resolutions 1 to 8  μm/px. If a slight performance decrease can be observed at higher resolutions, this is likely due to the larger influence of smaller structures that—due to the differences from slide to slide—lack a correspondence and that are otherwise invisible at coarser image resolutions.

Comparing the HyReCo to the ANHIR cases in Fig. 4, the overall MTRE is larger in the ANHIR dataset for all image resolutions ($p<0.001/N_{B}$ after Bonferroni correction by the number of image resolution pairs $N_{B}=6$).

#### 4.1.2 Restained sections compared to consecutive sections on the same tissue block

**Fig. 5:**
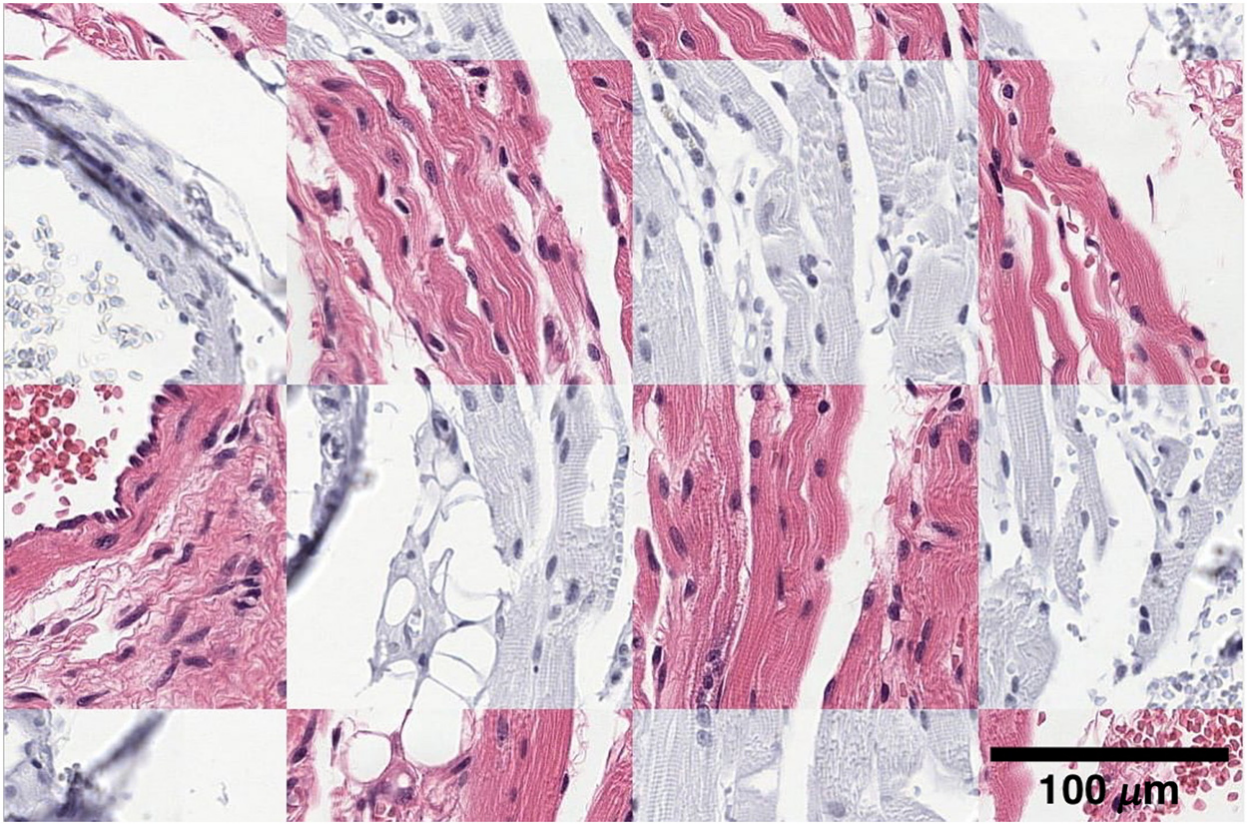
Checkerboard plot after registration of a restained image pair. Nucleus correspondences are visible at the borders of the checkerboard.

**Fig. 6:**
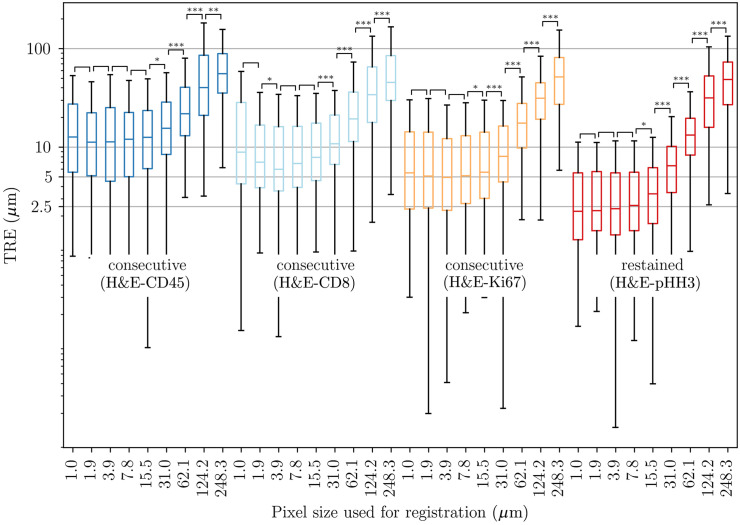
TRE after consecutive and restained deformable registration at different image resolutions of the HyReCo subset A for different staining pairs (logarithmic plot). The TRE between restained section (right group, red) is significantly lower than between consecutive sections (three left groups) for the respective best MTRE of all resolutions (p<0.001, respectively). The accuracy after registration of consecutive section depends on their distance (section order: H&E, Ki67, CD8, and CD45). The boxes denote the interquartile range and the whiskers extend this range by a factor of 1.5. Results of statistical tests between subsequent resolutions are given by [blank]: not significant and significant with *$p<0.05/N_{B}$, ***$p<0.01/N_{B}$, and *** $p<0.001/N_{B},$ after Bonferroni correction for $N_{B}=8$ image resolution pairs. N=138 for each boxplot.

**Fig. 7:**
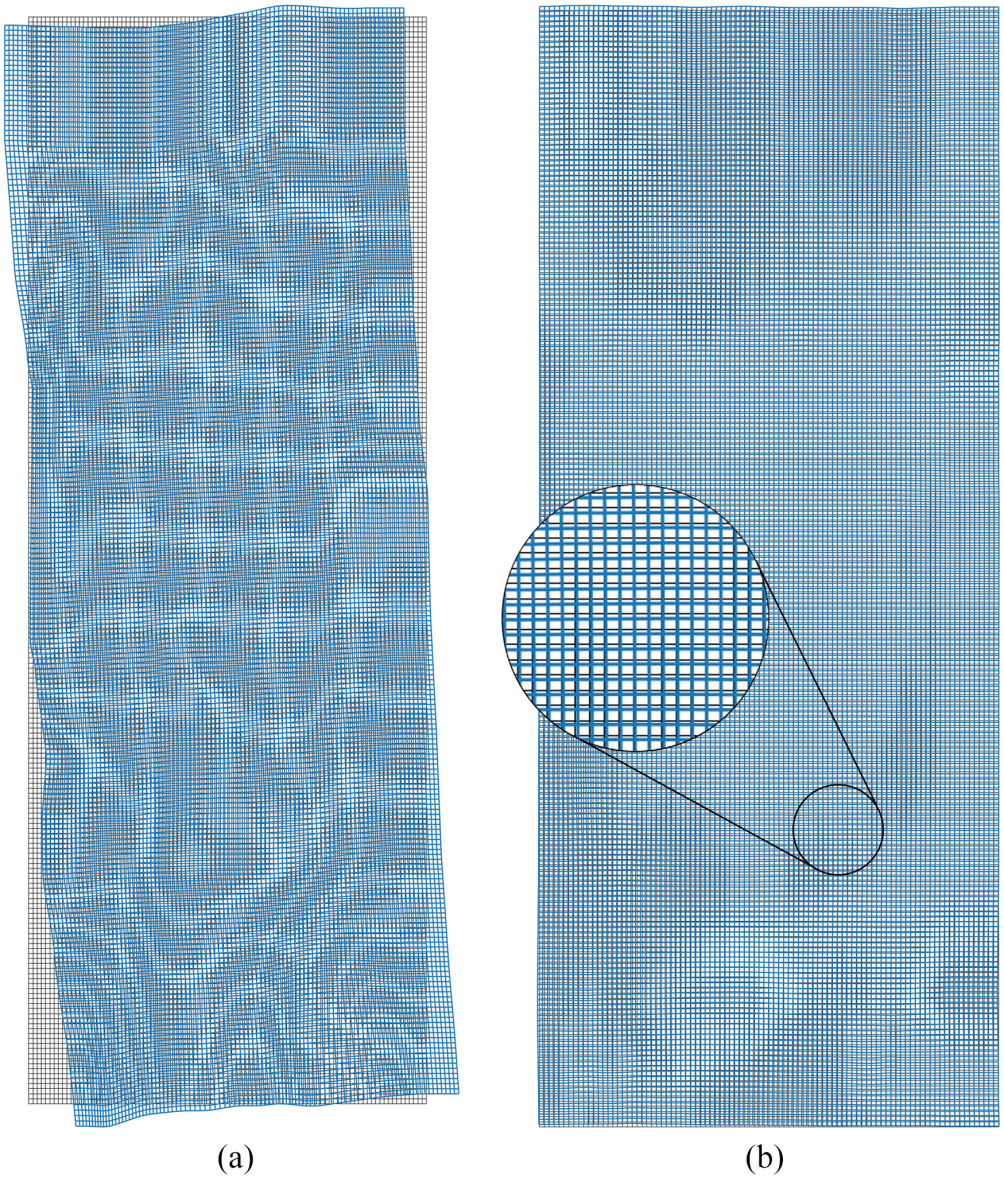
(a) Deformation of a consecutive and (b) a restained registration based on the same H&E-stained slide (image stack 361 of the HyReCo dataset). In each image, the deformation is applied to a regular grid (background, gray) and plotted in blue (foreground). The consecutive pair shows a larger nonlinear component, but small nonlinear effects are also visible between the two restained images.

A visual inspection of the registered restained sections shows very little differences (Fig. 5). The MTRE in the restained images in HyReCo subset A reaches 2.2  μm and is approximately two to four times lower than between consecutive sections (p<0.001, Fig. 6, Table 2). As expected, the deformation between consecutive image pairs shows stronger nonlinear components than between restained sections. No foldings were detected in the deformations in any of the restained image pairs. A visual comparison of the deformations after restained and after consecutive registrations is shown in Fig. 7.

**Table 2:** Best median TRE obtained and required image resolution. The MTRE between restained sections is lower by a factor of ∼2. MTRE increases with the distance between the sections for consecutive sections.

Pair of stains	Best MTRE (μm)	im. resolution (μm/px)
HE-pHH3 (restained)	2.2	1.0
HE-Ki76	4.9	3.9
HE-CD8	6.0	3.9
HE-CD45RO	11.2	1.9
All HyReCo consecutive	6.5	3.9
ANHIR	24.1	1.0

### 4.2 Experiment 2: Deformable Compared to Affine Registration

The two images of the restained section pair show the same tissue specimen before and after an additional chemical processing and scanning. We show that deformable registration leads to superior results despite the tissue being fixed at the glass slide during restaining. To this end, we compare the MTRE after affine and deformable registration in all datasets.

#### 4.2.1 Improved accuracy of deformable registration in all datasets

**Fig. 8:**
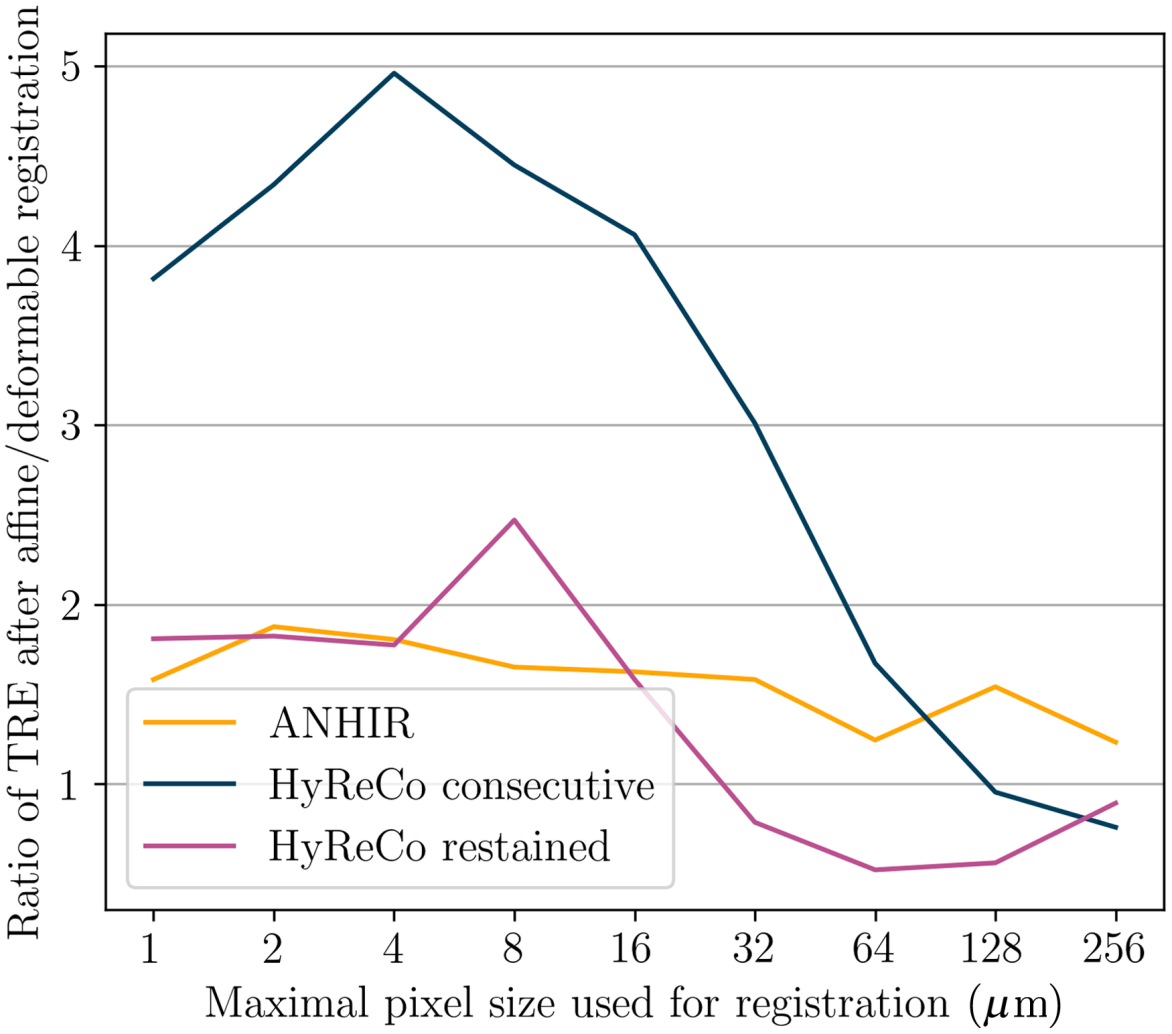
Ratio $\frac{\mathrm{MTRE}\;\text{affine}}{\mathrm{MTRE}\;\text{deformable}}$ for ANHIR and the consecutive and restained HyReCo datasets. Deformable registration outperforms affine registration except for image resolutions coarser than 16  μm/px.

Deformable registration outperforms affine registration except for image resolutions coarser than 16  μm/px in all datasets ($p<0.001/N_{B}$ after Bonferroni correction by the number of image resolution pairs $N_{B}=8$, Fig. 8, Table 3). The advantage is less expressed in restained sections where even affine registration leads to a low registration error (1.7  μm restained against 20.2  μm consecutive), which is due to the smaller mechanical deformation in the processing. The lower difference in the ANHIR dataset compared to the consecutive subset of HyReCo is likely due to the larger proportion of artifacts and structures without correspondence in this dataset.

**Table 3:** Best MTRE obtained after affine and deformable registration.

Pair of stains	MTRE affine (μm)	MTRE deformable (μm)
ANHIR	38.1	24.1
HyReCo consecutive (subset A)	20.2	5.3
HyReCo restained (subset B)	1.7	0.9

#### 4.2.2 Superiority of deformable registration in a separate, restained dataset

In the separate subset B of restained slides (H&E-PHH3) where no consecutive sections are available, the MTRE is lower and reaches 0.9  μm, which approaches the intraobserver error.

**Fig. 9:**
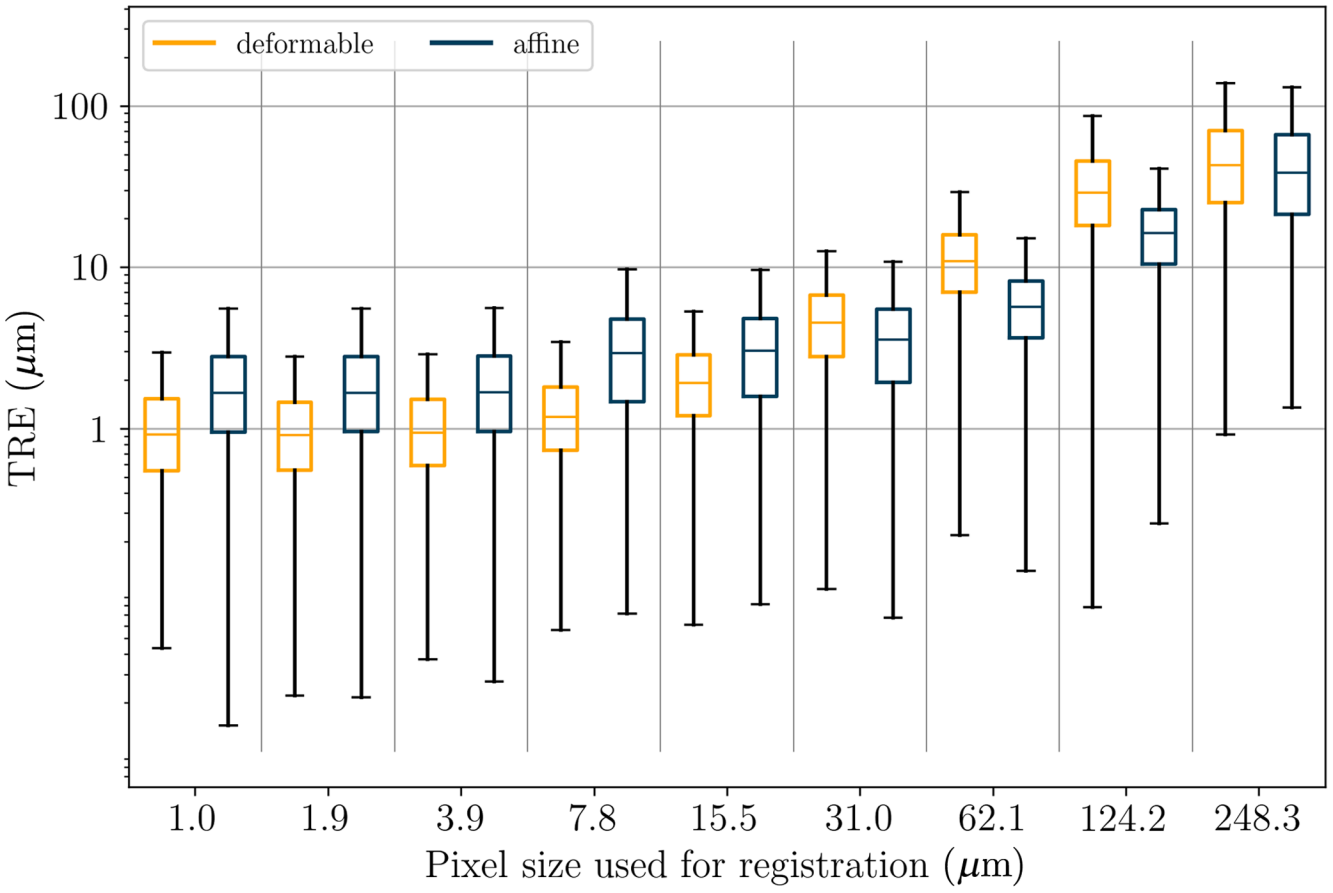
TRE of restained image pairs after affine and deformable registration at different image resolutions (HyReCo subset B). Deformable registration does further improve the landmark error if compared to affine registration for pixel sizes ≤31.0  μm ($p<0.001/N_{B}$ after Bonferroni correction by the number of comparisons $N_{B}=9$). For the deformable registration, the best results are obtained with a pixel size ≤1.9  μm ($p<0.001/N_{B}$ after Bonferroni correction by twice the number of image resolution pairs $N_{B}=16$). N=2357 for each boxplot.

In subset B, the deformable registration again lowers the landmark error compared to affine registration ($p<0.001/N_{B}$ after Bonferroni correction by the number of comparisons $N_{B}=9$, Fig. 9), but to a lower degree than between consecutive sections (0.9  μm compared to 1.7  μm). A visualization of the deformation field after restained section registration shows a small nonlinear component, which is consistent with the lower landmark error (Fig. 7).

### 4.3 Computation Times of Deformable Compared to Affine Registration

The computation time of an image registration algorithm depends largely on the implementation and on the size of the input images but also on other factors, such as CPU and RAM performance and disk access. We report the measurements of our setup (Intel^®^ Core™ i7-7700K CPU (4.20 GHz, four cores) with 32 GB of RAM) to give a relative comparison with respect to the size of the images. The implementation of the registration algorithm has been optimized for memory-efficient and parallel data handling as described in Ref. 30.

For an affine registration on the HyReCo data, (we do not systematically report the computation times in the ANHIR data because of a large number of different image sizes which makes the comparison of the computation times difficult.), the average computation time ranges from 0.5 s (image size 400×800, 62.1  μm/pixel) to 58 s (image size 12,800×25,600, 1.94  μm/pixel). The majority of the computation time for large images is spent on the deformable registration. Previous analyses^30^ have shown that doubling the image resolution (an increase of four times the number of pixels) leads to a fourfold increase in the computation time. In other words, the computation time is roughly linearly dependent on the number of pixels in the image. Together with the contributions from prealignment and affine registration, we see a similar trend in the computation times in Table 4. For deformable registration on the HyReCo data, the average computation time ranges from 2.6 s to 30 min.

**Table 4:** Execution time for a deformable registration of consecutive sections with respect to image size (and resolution). Larger image sizes require a larger computation time.

Image resolution (μm/px)	Mean execution time (s)	Approx. image size (px)
248.32	3.1	100 × 200
124.16	2.9	200 × 400
62.08	2.6	400 × 800
31.04	3.7	800 × 1600
15.52	8.3	1600 × 3200
7.76	24	3200 × 6400
3.88	89	6400 × 12,800
1.94	310	12,800 × 25,600
0.97	1831	25,600 × 51,200

## 5 Discussion

We compared the accuracy of numerical image registration at different image resolutions in restained and consecutive sections.

### 5.1 Image Resolution in Restained and Consecutive Sections

The accuracy of the registration depends on the employed image resolution. In our experiments, the impact of the image resolution was highest in restained sections where optimal results could be reached at an image resolution ≤1.9  μm/px. Finer image resolutions even exceeded 180 GB of RAM on a more powerful computer. With this accuracy, the restained sections allow a nucleus-level alignment that can be used for a multiplexed analysis of the finest structures in the image.

Similar results have been reported for registration of multiplexed immunofluorescence images in Refs. 6 and 34, where the authors report a median registration error of ca., 1 to 1.5  μm.

When comparing different image resolutions in consecutive sections, a smaller pixel size is correlated with a smaller registration error up to a level of saturation that differs between datasets. The gain in accuracy of the registration stagnates between 15.5 and 7.8  μm/px such that these registrations can be computed based on smaller image size and hence require less memory and time.

Different resolution levels are commonly used in multilevel image pyramids to align images while avoiding convergence to local minima.^13^ In theory, higher image resolution in two images showing the same structures should lead to a more accurate alignment, which is consistent with our observations in restained sections. To our knowledge, no systematic evaluation of the effect of increased image resolution has been done for registration in histopathology.

### 5.2 Slice Similarity in Consecutive Sections

The similarity between sections influences the registration accuracy and differs between the ANHIR and HyReCo datasets as well as in the literature. To relate to the values reported in the ANHIR challenge,^17^ we recomputed the original pixel sizes and again observe a large difference between the individual subdatasets (see Fig. S1 in the Supplementary Material). Also Xu et al.^35^ found a large variety in the registration error in prior work on histology 3D reconstruction.

In the recently published results of the ACROBAT challenge,^36^ the annotators obtain a median interobserver error of 21  μm. Considering the interobserver error of 1.1  μm in the HyReCo dataset, the higher interslice similarity seems to be the main driver of registration accuracy.

Some studies report TREs that are similar to this study. In Ref. 37, the authors recommend a pixel spacing similar to the interslide distance as a starting point. Similar to our results, they do not find a clear advantage of a pixel size of 4  μm/px compared to 8  μm/px. Kiemen et al.^38^ obtained an absolute median TRE of 3.5  μm at an image resolution of 8  μm/px, which is similar to our registration results of directly neighboring slides at this resolution.

The similarity between sections decreases as the distance between two consecutive sections increases. Small structures cannot be aligned if their counterparts are not present in the other slide.

From the landmark errors in consecutive sections (Fig. 6), we are able to derive the likely section order (HE, Ki67, CD8, and CD45RO) as the distance between two sections in the stack grows, the landmark error increases as well. The registration accuracy in consecutive sections largely depends on the quality and similarity of the sections.

In the previous work, section distance was identified as the main factor affecting registration accuracy in 3D reconstruction of histological sections ^38^ [see source data for Figs. 2(a)–2(d)]. We observe similar effects when comparing the registration of slide pairs with different distances (Table 2 and Fig. 4).

We suspect that missing correspondences caused by larger slice distances are the cause of the saturation of registration accuracy at finer image resolutions, where fine structures can no longer be matched.

### 5.3 Restained Compared to Consecutive Section Registration

Restained sections do hardly suffer from the problem of missing correspondences. The difference in the alignment quality between restained and consecutive sections is relevant for applications where small structures or single nuclei are of interest. An MTRE of 1.0  μm allows nucleus-level alignment, which is infeasible in serial sections where the same nucleus is often not present on the next slide. For comparison, the size of an average mammalian nucleus is ∼6  μm,^39^ and tissue sections typically measure 2 to 5  μm thick. The increased accuracy is at the expense of losing the physical slide and increasing processing time by destaining. Only the staining that is applied last can be conserved physically. Especially in clinical settings, long-term storage of the glass slides and short time to diagnosis are important. Both physical slides can be preserved when using consecutive sections for registration. The lower accuracy is sufficient for aligning larger areas, such as tumor or inflammatory areas, which are often needed to define a region of interest for an analysis carried out in a differently stained neighboring section. ^40^

Even in the restaining process, smaller nonlinear deformations occur, probably due to mechanical and chemical manipulation and tile stitching during scanning. These nonlinear components can also be observed in the deformation fields resulting from the registration of restained images. When aiming for high registration accuracy in restained images, deformable image registration further reduces the landmark error at fine image resolutions. This observation is consistent with common image registration practice, which often uses a multistep approach with increasing degrees of freedom in each step.^13^^,^^17^

### 5.4 Limitations

The median landmark error in restained sections is lowered to 0.9  μm. When compared on the same block of tissue, the registration error between restained sections is a factor of two to five lower than the corresponding consecutive sections (2.2  μm compared to 6.5  μm). The difference between subsets A and B is likely influenced by the pairwise landmark setup. In consecutive sections, the accuracy depends largely on the quality of the sections and the image resolution. When comparing the two datasets, the larger landmark error between the ANHIR images is likely due to the larger structural differences between the slides in some of its subsets (Fig. 3). We note that the registration parameters were optimized on the ANHIR dataset, which may bias the results in its favor.

Our analysis is limited by the focus on landmarks as the only measurement of accuracy. Since the landmarks were placed at positions that can be reidentified by a human observer, these locations likely have a superior contrast and thus have a higher impact on the distance measure. This could lead to a bias in the evaluation that underestimates the registration error in larger, low-contrast regions.

Especially in low-contrast regions, the regularizer maintains the smoothness of the deformation. The regularity or smoothness of the deformation is also a quality criterion for an image registration. We automatically analyzed the deformed grid for folds (no occurrences in HyReCo subset A and B) but otherwise did not systematically evaluate smoothness of the deformation except for visual inspection.

As previously mentioned, the discrepancies in registration errors between the ANHIR and HyReCo datasets are likely a result of different intersection similarity. We were not able to further analyze additional factors, such as patient demographics, dataset age, or scanning hardware, as this information is not available.

## 6 Conclusion

Restained sections allow an accurate registration of differently stained structures that is below the level required to align single nuclei. Registrations of consecutive sections result in a higher alignment error that increases with the distance between the slides. Consecutive sections are better suited to align larger areas, such as tumor or inflammatory areas, based on a second stain. We recommend deformable registration that was always more accurate, and the use of restained sections, if possible. Higher image resolutions benefit the accuracy, as long as the increase in image detail leads to an increase in corresponding structures.

## 7 Appendix A: Image Registration

Given a so-called reference image $R:R^{2}\to R$ and a so-called template image $T:R^{2}\to R$, the goal of image registration is to find a reasonable spatial transformation $y:R^{2}\to R^{2}$ such that R(x)≈T(y(x)), i.e., R and the deformed template T∘y are similar in an adequate sense.

Following Ref. 13, we formulate the image registration as the optimization problem $J(R,T,y)\overset{y}{\to }\min$ of an appropriate objective function J with respect to the desired spatial transformation. A key component of the objective function is a so-called distance or image similarity measure that quantifies the quality of the alignment. We use the NGF distance measure^31^ as it has been shown to be robust to different stains and is suitable for multimodal image registration of histological images.^4^ For the discretization, 2D images with extents $n_{1}\times n_{2}$ are assumed, correspondingly consisting of N=n_1·n_2  pixels with uniform size h∈R in each dimension and pixel centers $x_{1},\ldots ,x_{N}$; $x\_i\in R^{2}$. The NGF distance measure is given by

$$\mathrm{NGF}(R,T,y)=\;\frac{h^{2}}{2}\cdot \overset{N}{\underset{i=1}{\sum }}1-{\left(\frac{{\left\langle \nabla T(y(x_{i})),\nabla R(x_{i})\right\rangle }_{\varepsilon }}{{\left\|\nabla T(y(x_{i}))\right\|}_{\varepsilon }{\left\|\nabla R(x_{i})\right\|}_{\varepsilon }}\right)}^{2},$$

with ${\left\langle x,y\right\rangle }_{\varepsilon }=x^{⊺}y+\varepsilon^{2}$, ${\left\|x\right\|}_{\varepsilon }:=\sqrt{{\left\langle x,x\right\rangle }_{\varepsilon }}$, and the edge parameter ε, which controls the sensitivity to edges in contrast to noise. This image distance becomes minimal if intensity gradients and edges, respectively, are aligned and that therefore leads to the alignment of morphological structures.

The NGF distance measure was used in all three steps of the registration pipeline: prealignment, affine registration, and deformable (nonlinear) registration. In addition, we used a multilevel optimization scheme that starts with the registration of images at low-resolution levels and then refines the transformation to higher image resolutions. This stepwise approach reduces the risk of converging too early to local minima and accelerates the optimization process.^41^ The per-level optimization was performed using a Gauss–Newton type (affine registration) and L-BFGS quasi-Newton (deformable registration) method (see e.g., References 13 or 42 and 43) for a more detailed discussion and additional strategies.

The NGF distance measure is based on structural changes expressed through the image gradient, and therefore, color information is of limited value. To reduce the amount of image data to be handled, all images are converted from color to gray scale and inverted to obtain a black background while loading from disk.

Images are assumed to be available in a multilevel image data format to reduce the time and memory requirements to load the image data at a given resolution.

### 7.1 Step 1: Automatic Rotation Alignment

Before histological images were scanned, the tissue was cut, preprocessed, and stained in a pathology lab.

After this manual process, a part of the neighboring tissue slices can end up in arbitrary positions on the object slide (such as upside down or turned in various ways). In general, no assumptions can be made on the initial tissue positioning and—in a first step—we aim to find a rigid alignment, correcting for global translation and global rotation.

Automatic rotation alignment first determined the center of mass^32^ of both images, using the gray values of the pixels as the weights. Let $\left(t_{1},t_{2}\right)$ be the vector pointing from the center of mass of the reference image to the center of mass of the template image, and let $\phi_{k}=2\pi (k-1)/(N_{\text{rotations}}-1)$, $k=1,\ldots ,N_{\text{rotations}}$ be equidistant rotation angles sampling the interval [0,2π). For each angle, a rigid registration was computed, optimizing:

$$J(R,T,y_{\text{rigid}})=\mathrm{NGF}(R,T,y_{\text{rigid}})\to \min,>y_{\text{rigid}}:R^{2}\mapsto R^{2},y_{\text{rigid}}\;\text{parameterizedby}(\phi_{k},t_{1},t_{2}),$$

with initial parameters $\left(\phi_{k},t_{1},t_{2}\right)$, $k=1,\ldots ,N_{\text{rotations}}$. Among all $N_{\text{rotations}}$ rigid registration results, the minimizer $y_{\text{rigid}}^{*}$ with the smallest image distance was selected as an initial guess for the subsequent affine registration.

### 7.2 Step 2: Affine Registration

In a second step, again an NGF-based image registration was computed. To allow for additional degrees of freedom, the registration was optimized with respect to an affine transformation $y_{\text{affine}}$ and based on a finer image resolution with the previously computed $y_{\text{rigid}}^{*}$ as an initial guess. The resulting transformation was then used as initial guess for a subsequent deformable registration.

### 7.3 Step 3: Deformable Registration

The final step was a deformable image registration. Here the transformation y is given by

y(x)=x+u(x),

with so-called displacement $u:R^{2}\to R^{2}$, $u=(u_{1},u_{2})$.^13^

In contrast to an affine registration, the deformation is not restricted to a particular parameterizable deformation model. The nonlinear transformation is controlled by introducing a regularization term into the objective function that measures the deformation energy and penalizes unwanted transformations. Here, we used the so-called curvature regularization, which penalizes second-order derivatives of the displacement^44^ and which has been shown to work very well in combination with the NGF distance measure.^29^^,^^30^ As with the NGF distance, we evaluated the displacements in the pixel centers $x_{1},\ldots ,x_{m}$ with uniform grid spacing h and used finite differences to approximate the derivatives. Thus the discretized curvature regularizer is defined as

$$\mathrm{CURV}(y)=\frac{h^{2}}{2}\overset{m}{\underset{i=1}{\sum }}{\left|\Delta^{h}u_{1}(x_{i})\right|}^{2}+{\left|\Delta^{h}u_{2}(x_{i})\right|}^{2},$$

where $\Delta^{h}$ is the common five-point finite difference approximation of the 2D Laplacian $\Delta =\partial_{xx}+\partial_{yy}$ with Neumann boundary conditions. In summary, for deformable registration, we minimize the objective function:

J(R,T,y)≔NGF(R,T,y)+αCURV(y)→min,

with respect to the deformation y with the previously computed optimal $y_{\mathrm{affine}}$ as initial guess. The parameter α>0 is a regularization parameter that controls the smoothness of the computed deformation. The parameter α is chosen manually to achieve a smooth deformation and avoid topological changes (lattice folds), while being flexible enough to correct for local changes that improve image similarity. The resolution of the control point grid is independent of the image resolution and is typically set to be coarser than the image resolution (see also Table 1). A higher number of grid points allows for a more accurate representation of local deformations. Linear interpolation is used to evaluate the deformation between its grid nodes.

## 8 Appendix B: Evaluation

Figure S1 in the Supplementary Material shows the TRE after registration of the different ANHIR tissue types. As already reported in the results of the ANHIR challenge,^17^ the dataset is heterogeneous in tissue quality and image resolution, which is also reflected in the TREs.

## Data Availability

The HyReCo image data that were used in this study are openly available in IEEE dataport at https://dx.doi.org/10.21227/pzj5-bs61.
