# Supplementary material for: Comparison of consecutive and restained sections for image registration in histopathology
Source: J Med Imaging (Bellingham). 2023 Nov 30;10(6):067501. doi: 10.1117/1.JMI.10.6.067501 (PMC10704256; doi:10.1117/1.JMI.10.6.067501)
Supplement: Supplementary file 1 [file JMI_010_067501_SD001.pdf]

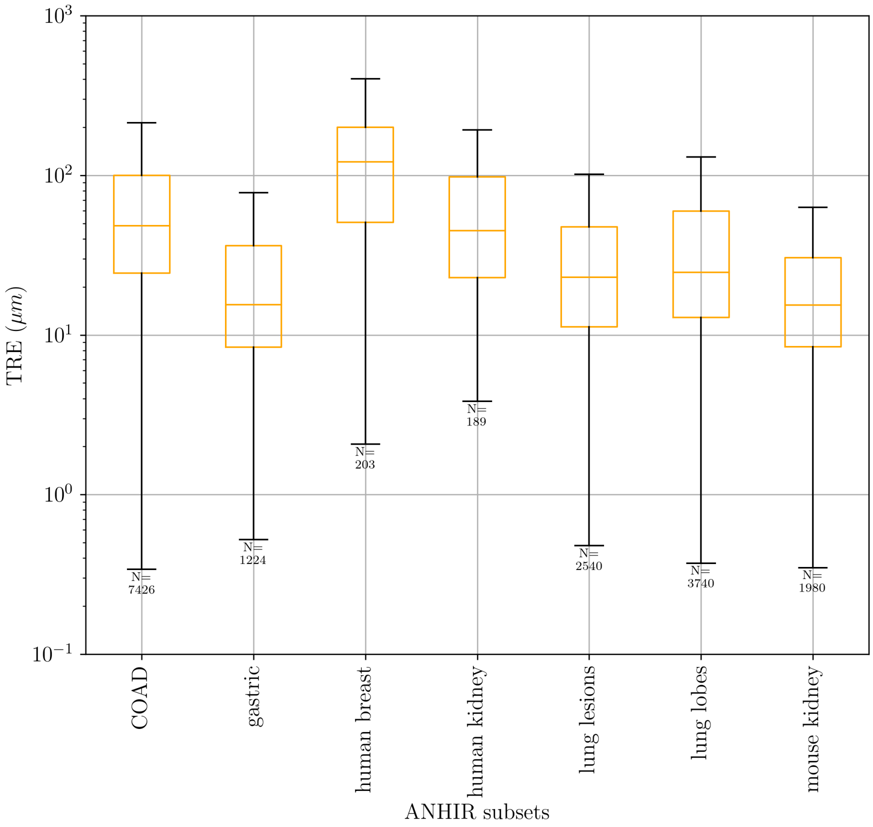

Supplementary Figure 1 TRE after deformable registration of the different tissue types of the ANHIR dataset. Images have been registered at a standardized size of max. 4000 pixels in each dimensions as image resolution differs between tissue types. The ANHIR dataset is heterogeneous in terms of tissue quality and resolution which is also reflected in the registration result. This is consistent with the varying results for different tissue types reported in the ANHIR challenge paper.
